# Communication interventriculaire post infarctus du myocarde: réparation chirurgicale

**DOI:** 10.11604/pamj.2014.19.68.4284

**Published:** 2014-09-24

**Authors:** Hicham Labsaili, Said Makani

**Affiliations:** 1 Service de Chirurgie Cardiovasculaire, CHU Ibn Rochd, Casablanca, Maroc

**Keywords:** Communication interventriculaire, infarctus du myocarde, occlusion de l′IVA, l′artère interventriculaire antérieure (IVA), Ventricular septal defect, myocardial infarction, occlusion of the coronary artery, anterior interventricular the artery

## Image en medicine

La communication interventriculaire (CIV) post infarctus du myocarde est une complication aiguë.l'incidence de cette complication est évaluée entre 1% et 2%, mais elle est responsable de 5% des décès en phase aiguë de l'infarctus. Sa prise en charge est chirurgicale, avec la difficulté de réparation à partir des tissus infarcis fragiles. Le septum interventriculaire a une double vascularisation, ce qui explique les deux types de localisation de CIV: CIV antérieure par occlusion de l'artère interventriculaire antérieure (IVA), qui est la plus fréquente, et la CIV postérieure par occlusion d'une coronaire droite dominante ou plus rarement d'une circonflexe dominante. Nous rapportons le cas d'une réparation chirurgicale, faite avec succès, d'une CIV antérieure de 15mm, chez un patient de 75 ans, qui avait une occlusion de l'IVA. La réparation a été faite sous circulation éxtracorporelle, avec résection des tissus infarcis et mise en place d'un patch adapté au défect. Le patch a été positionné du coté ventriculaire gauche. Des points en U sur feutre de Teflon ont fixé le patch. Les suites opératoires ont été favorables et le patient est sorti du bloc opératoire avec de faible dose de DOBUTAMINE.

**Figure 1:**
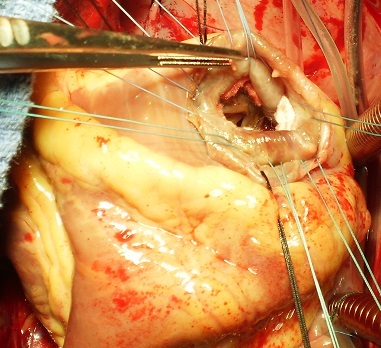
Vue opératoire de la communication interventriculaire apicale, avec passage des fils en points en U renforcés par du feutre de Téflon

